# Long-term clinical experience with a 3-step combined technique for involutional lower eyelid entropion and ectropion based on common pathophysiologic mechanisms

**DOI:** 10.3389/fsurg.2026.1892004

**Published:** 2026-09-29

**Authors:** A. Pasteris, M. Rodio, S. Rampazzo, F. Ziani, E. Trignano, C. Rubino

**Affiliations:** 1 Plastic, Reconstructive and Aesthetic Surgery Training Program, University of Sassari, Sassari, Italy; 2 Department of Medicine, Surgery and Pharmacy, University of Sassari, Sassari, Italy; 3 Plastic Surgery Unit, University Hospital Trust of Sassari, Sassari, Italy

**Keywords:** combined procedure, involutional ectropion, involutional entropion, lower eyelid malposition, wedge resection

## Abstract

**Background:**

Involutional lower eyelid malpositions, including ectropion and entropion, are common age-related conditions resulting from horizontal eyelid laxity, imbalance between the anterior and posterior lamellae, and orbicularis oculi muscle dysfunction. Although numerous surgical techniques have been described, no single approach has proven universally superior. At our institution, two previously described condition-specific 3-step procedures are used to address the specific pathophysiologic mechanisms underlying involutional ectropion and entropion.

**Methods:**

A retrospective analysis was conducted on patients treated between 2012 and 2025 using condition-specific 3-step surgical techniques. For involutional ectropion, the procedure combined lower eyelid skin release, lateral eyelid shortening through a base-up full-thickness triangular resection, and transposition of a laterally based upper eyelid myocutaneous flap. For involutional entropion, the technique consisted of partial orbicularis oculi muscle myectomy, lateral eyelid shortening through a base-down triangular resection, and excision of a lower eyelid skin strip. Functional and aesthetic outcomes, postoperative complications, and recurrence rates were evaluated throughout follow-up.

**Results:**

Forty-five patients (57 eyelids) underwent ectropion correction, with a mean follow-up of 22 months. The overall success rate was 94.7%, with one case of undercorrection and two late recurrences, all successfully managed with secondary procedures. Complications were minimal and limited to one early wound dehiscence. Thirty-five patients (40 eyelids) underwent entropion correction, with a median follow-up of 12 months. No recurrences were observed, while one case of undercorrection and one early wound dehiscence were reported.

**Conclusions:**

Involutional ectropion and entropion share a common pathophysiologic substrate characterized by horizontal eyelid laxity, lamellar imbalance, and orbicularis oculi dysfunction. Despite technical differences, both procedures are based on the same reconstructive principles: restoration of horizontal eyelid tension, rebalancing of the anterior and posterior lamellae, and normalization of orbicularis muscle forces. The favorable long-term outcomes and low recurrence rates observed in both groups support a pathophysiology-driven, vector-based reconstructive approach for the management of involutional lower eyelid malpositions.

## Introduction

Involutional lower eyelid malpositions represent one of the most common age-related disorders affecting the periocular region. Among these conditions, involutional ectropion and entropion are frequent clinical entities that significantly impact ocular comfort, function, and quality of life, particularly in the elderly population (1). Involutional ectropion is characterized by an outward rotation of the eyelid margin secondary to age-related degeneration of the structural and functional components of the eyelid, whereas involutional entropion is defined by an inward rotation of the eyelid margin with subsequent contact between the eyelashes and the ocular surface (2). Both conditions are associated with substantial morbidity and may lead to chronic ocular irritation, epiphora, and visual discomfort (3).

The clinical consequences of involutional ectropion include exposure keratopathy, conjunctival inflammation, recurrent infections, punctal malposition, and persistent tearing, all of which may significantly affect patients’ quality of life and psychological well-being. Similarly, involutional entropion may result in foreign body sensation, chronic irritation, corneal epithelial damage, and in severe cases corneal scarring and visual loss. Epidemiologically, involutional ectropion affects approximately 2.9% of individuals over 60 years of age, while involutional entropion has an estimated prevalence of 2.1% in the elderly population, with incidence increasing with age for both conditions (4).

Despite their opposite clinical presentation, both disorders arise from a shared pathophysiologic substrate. Involutional lower eyelid malposition is fundamentally a multifactorial condition resulting from progressive horizontal eyelid laxity and mechanical imbalance between the anterior and posterior lamellae. The main etiologic factors include attenuation of the tarsoligamentous support system, loosening of the tarsus, laxity or disinsertion of the canthal tendons, and weakening of the lower eyelid retractors, all of which contribute to horizontal and vertical instability of the eyelid (5).

A key role is also played by the degeneration and atrophy of the orbicularis oculi muscle, which compromises the dynamic stability of the lower eyelid. In involutional entropion, overriding of the preseptal orbicularis oculi muscle over the pretarsal portion generates an inward rotational vector that displaces the eyelid margin toward the globe. Additional contributing factors, such as orbital fat prolapse and age-related changes in periocular soft tissues, may further enhance this inward force. In involutional ectropion, by contrast, loss of orbicularis tone, anterior lamellar contracture, horizontal eyelid elongation, and gravitational midface descent contribute to an outward rotational vector, resulting in eyelid eversion and punctal malposition (6). Over time, chronic exposure leads to secondary inflammatory changes, tarsal thickening, and cicatricial alterations that further perpetuate the malposition, establishing a self-reinforcing pathological cycle (7).

Successful surgical correction of involutional lower eyelid malpositions requires a comprehensive understanding of eyelid biomechanics and a multi-vector approach addressing all contributing factors. Numerous surgical techniques have been described for the treatment of both ectropion and entropion, including lateral tarsal strip procedures (8–10), wedge resections, retractor reinsertion (11), everting sutures (12), orbicularis muscle modifications, and various skin and canthal tightening techniques (13) (14). However, no single procedure has demonstrated universal superiority, largely due to the multifactorial nature of these conditions.

To overcome these limitations, combined procedures targeting multiple etiologic components have been proposed. The individual surgical procedures evaluated in the present study were previously described by our group for the treatment of involutional ectropion and entropion, respectively (15, 16). The present study should therefore not be considered a first description of either surgical technique. Rather, it represents an expanded and updated clinical analysis of these procedures, incorporating additional patients and updated follow-up of the previously reported cohorts.

Although technically distinct, both procedures are designed to restore horizontal eyelid tension, rebalance the anterior and posterior lamellae, and normalize orbicularis oculi muscle dynamics through correction of pathological vector forces.

The aim of the present study is therefore to provide an expanded and updated clinical evaluation of two previously described, condition-specific three-step procedures and to examine their shared reconstructive rationale. The present study does not seek to compare involutional ectropion and entropion as equivalent clinical entities, nor to establish superiority of one procedure over the other. Instead, it investigates whether a common pathophysiology-driven framework, based on restoration of horizontal eyelid tension, rebalancing of the anterior and posterior lamellae, and modulation of orbicularis-related forces, can provide a coherent basis for the surgical management of these two opposite manifestations of lower eyelid involution. The current analysis includes additional patients beyond the previously reported cohorts and updated follow-up of previously reported patients, thereby extending the clinical experience associated with both techniques.

## Materials and methods

This retrospective study analyzed the medical records of patients who underwent surgical correction of involutional lower eyelid malpositions using condition-specific 3-step techniques at the Plastic Surgery Unit, Azienda Ospedaliera-Universitaria di Sassari, Sassari, Italy, between 2012 and 2025.

All procedures included in the present cohort were performed by the same senior oculoplastic surgeon, with assistance from a junior surgeon throughout the study period (2012–2025). The senior surgeon was responsible for preoperative assessment, surgical planning, and performance of the operative procedure, while the junior surgeon provided surgical assistance.

The present study includes patients who were also represented in our previously published series describing the individual three-step procedures for involutional ectropion and entropion (15, 16), together with additional patients treated after the respective previous study periods. For ectropion, the previous series included 36 patients involving 45 eyelids, whereas the present cohort includes 45 patients involving 57 eyelids, comprising the previously reported patients and 9 additional patients involving 12 additional eyelids. For entropion, the previous series included 27 patients involving 30 eyelids, whereas the present cohort includes 35 patients involving 40 eyelids, comprising the previously reported patients and 8 additional patients involving 10 additional eyelids. Follow-up of patients included in the previous series was updated for the present analysis. The additional patients in the entropion cohort had 12 months of postoperative follow-up.

The present study therefore represents an expanded and updated clinical analysis rather than a first description of either surgical technique. The relationship between the previously published cohorts and the current study is summarized in Table 1.

**Table 1:** Comparison between previously published cohorts and the present study.

Population cohorts	**Previous ectropion study**	**Present ectropion cohort**	**Previous entropion study**	**Present entropion cohort**
Patients	36	45	27	35
New patients	—	9	—	8
Eyelids	45	57	30	40
Surgical technique	Same 3-step procedure	Same	Same 3-step procedure	Same
Follow-up	22 months mean	22 months mean	12 months median	12 months median
Main contribution	Initial clinical series	Expanded cohort + unified framework	Initial clinical series	Expanded cohort + unified framework

Patients were divided into two cohorts based on diagnosis:

- involutional lower eyelid ectropion group
- involutional lower eyelid entropion group

All patients had a clinical diagnosis of involutional lower eyelid malposition (ectropion or entropion), defined as symptomatic eyelid margin displacement associated with horizontal eyelid laxity.

Inclusion criteria for both groups included:

- symptomatic involutional ectropion or entropion;
- horizontal eyelid laxity with a lid distraction test > 6 mm;
- delayed or reduced snap-back test indicating orbicularis oculi dysfunction.

Exclusion criteria included:

- cicatricial, paralytic, congenital, or spasmodic eyelid malpositions;
- history of trauma or previous surgery involving the periocular region;
- previous eyelid malposition surgery using alternative techniques;
- inflammatory or neoplastic periocular disease.

All three inclusion criteria were required for eligibility. Patients had to have (1) symptomatic involutional ectropion or entropion, (2) horizontal lower eyelid laxity demonstrated by a distraction test >6 mm, and (3) delayed or reduced snap-back indicating impaired dynamic eyelid function. Patients who did not meet all three criteria were not included in the study.

Demographic data including age and sex were recorded for all patients.

Preoperative evaluation included documentation of eyelid position, severity grading, and associated symptoms.

For the ectropion group, severity was classified (17) as:

- Grade I: scleral show with eyelid eversion;
- Grade II: visible conjunctival exposure;
- Grade III: severe eversion with fornix exposure.

Horizontal laxity was assessed by lower eyelid distraction from the globe, with values >6 mm considered pathological. Orbicularis oculi muscle function and lid stability were evaluated using the snap-back test.

No formal severity grading system was applied to the entropion cohort. Entropion severity was assessed clinically according to the degree of inward rotation of the eyelid margin, presence of lash–globe contact, associated ocular symptoms, and the functional eyelid laxity tests described above. A categorical grading system analogous to that used for ectropion was not retrospectively applied.

Surgical success was defined as restoration and maintenance of the intended physiological eyelid position, including normal eyelid–globe apposition in the ectropion group and restoration of the normal outward orientation of the eyelashes without lash–globe contact in the entropion group.

Secondary outcomes included:

- functional improvement (resolution of symptoms such as epiphora, irritation, foreign body sensation);
- aesthetic outcome (normal eyelid contour and symmetry with the contralateral side);
- complications;
- recurrence rates.

Undercorrection and overcorrection were defined as persistent or new eyelid malposition identified within 12 months postoperatively or earlier. Recurrence was defined as reappearance of eyelid malposition beyond 12 months after surgery.

Complications were classified as:

- early (within 2 weeks): hematoma, infection, edema, suture-related irritation, wound dehiscence, transient discomfort;
- late (after 2 weeks): undercorrection, overcorrection (ectropion or entropion depending on group), scar contracture, eyelid notching, chronic ocular surface irritation, and recurrence.

Functional assessment focused on symptom resolution, while aesthetic evaluation included eyelid margin position, absence of contour irregularities, and symmetry with the contralateral eyelid.

All data were recorded and analyzed using Microsoft Excel 2021. Continuous variables are presented as median values with corresponding ranges, whereas categorical variables are reported as absolute numbers and percentages.

### Surgical techniques

Local anaesthesia was administered using 2% lidocaine with 1:100,000 epinephrine injected with a 25-gauge needle into the inferior eyelid, lateral canthal region, and subconjunctivally into the lateral lower eyelid fornix to ensure adequate analgesia and a bloodless surgical field. The surgical techniques adopted in the present study were previously described by C. Rubino et al. (15, 16) and represent the standardized procedures currently employed at our Plastic Surgery Unit for the treatment of involutional lower eyelid malpositions.

Representative intraoperative photographs of the surgical steps were provided in the original publications describing these techniques (15, 16). In the present study, schematic illustrations are used to summarize the principal operative steps and facilitate their visualization without duplicating previously published photographic material.

Pre-operative markings were performed with the patient in the upright position to optimize symmetry and surgical planning. For ectropion correction, a horizontal line was marked approximately 2 mm below the lower eyelid margin, a laterally based orbicularis oculi myocutaneous flap was designed on the upper eyelid, with the inferior incision placed within the supratarsal crease approximately 8 mm from the ciliary margin, adjusted according to individual anatomy and refined using the pinch technique to avoid excessive tissue removal. The lower eyelid was approached through a subciliary incision along the marked line, followed by subcutaneous dissection and release of skin–orbicularis adhesions using Westcott scissors (Figure 1). Horizontal laxity was corrected by performing a full-thickness base-up triangular resection at the lateral canthus; after an initial incision approximately 2 mm from the lateral canthal angle, the eyelid was laterally distracted to tailor the extent of resection to the degree of laxity (Figure 2). The tarsus was then closed with a continuous 5-0 nylon suture, and the orbicularis muscle was reapproximated using buried interrupted 5-0 Vicryl Rapid sutures. The upper eyelid myocutaneous flap was dissected with careful preservation of the lateral palpebral vessels and marginal arcade, transposed to the lower eyelid, and secured with interrupted 5-0 nylon sutures (Figure 3), while the donor site was closed primarily with interrupted 5-0 nylon sutures (Figure 4) At the end of the procedure, tobramycin 0.3% ointment was applied, and the eye was patched for protection.

- Step 1 – Anterior lamellar release
- Step 2 – Base-up wedge resection
- Step 3 – Upper eyelid myocutaneous flap

**Figure 1:**
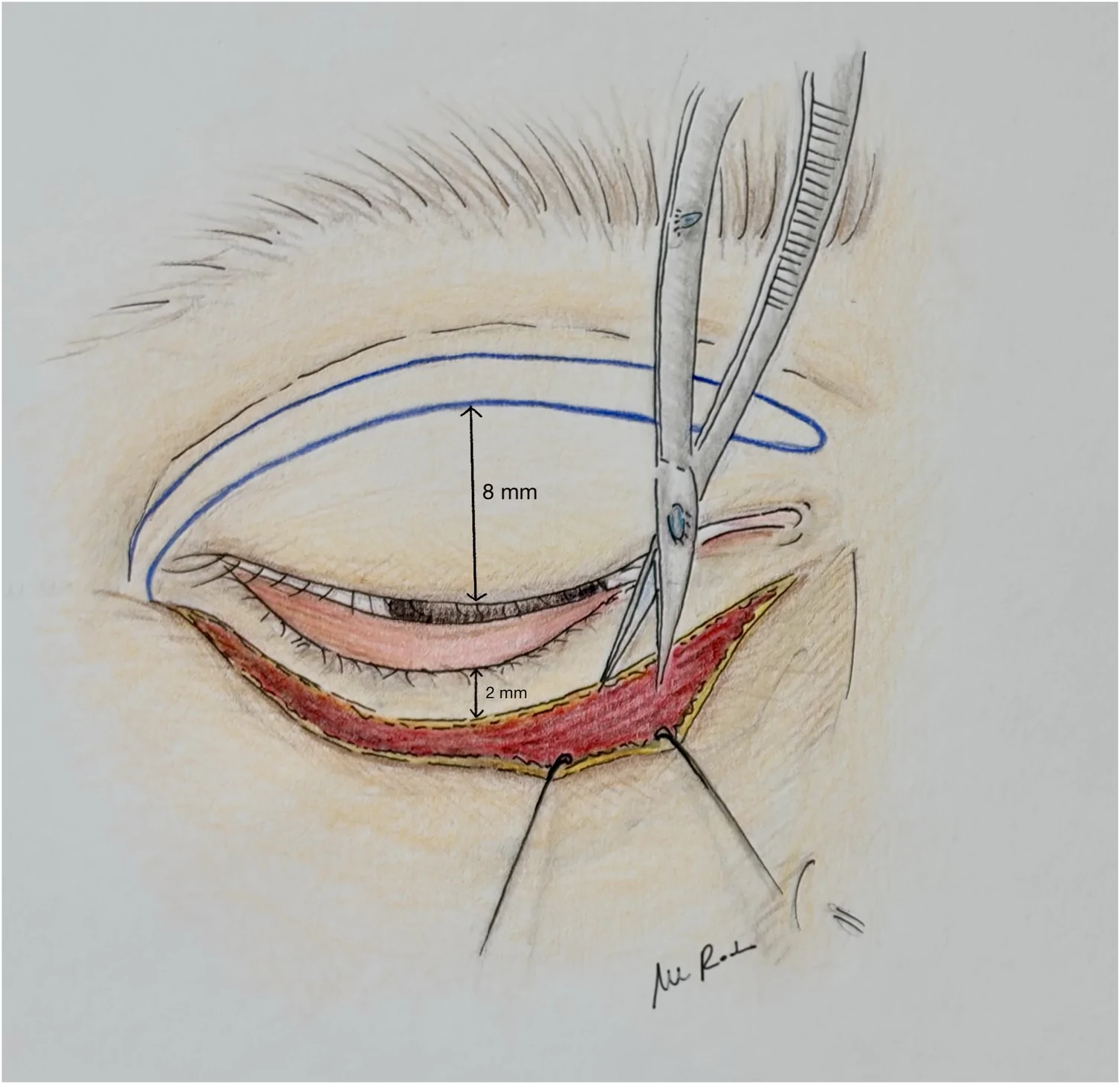
Preoperative planning and subciliary dissection for lower eyelid ectropion correction. Preoperative markings performed in the upright position and initial surgical approach. A horizontal incision line is marked 2 mm below the lower eyelid margin. Subcutaneous dissection and release of skin–orbicularis adhesions are carried out through the subciliary incision using Westcott scissors. On the upper eyelid, the inferior boundary of the laterally based orbicularis oculi myocutaneous flap is delineated within the supratarsal crease, 8 mm from the ciliary margin.

**Figure 2:**
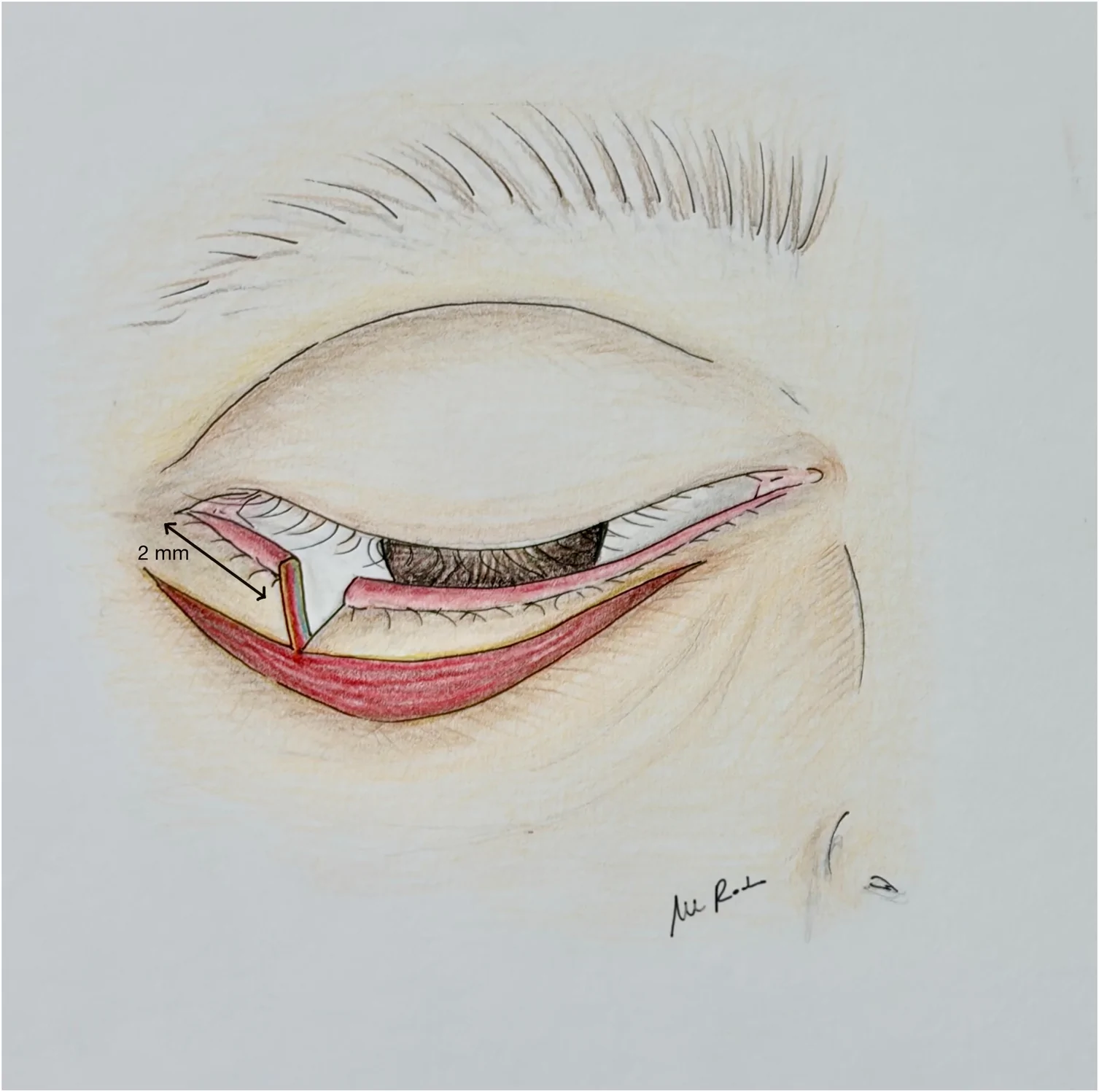
Correction of lower eyelid horizontal laxity via full-thickness lateral triangular resection. Intraoperative view detailing the management of eyelid laxity. Following an initial incision approximately 2 mm from the lateral canthal angle, the lower eyelid is distracted laterally to determine the exact extent of tissue redundant. A full-thickness, base-up triangular resection is performed at the lateral canthus to restore optimal tarsal tension prior to closure.

**Figure 3:**
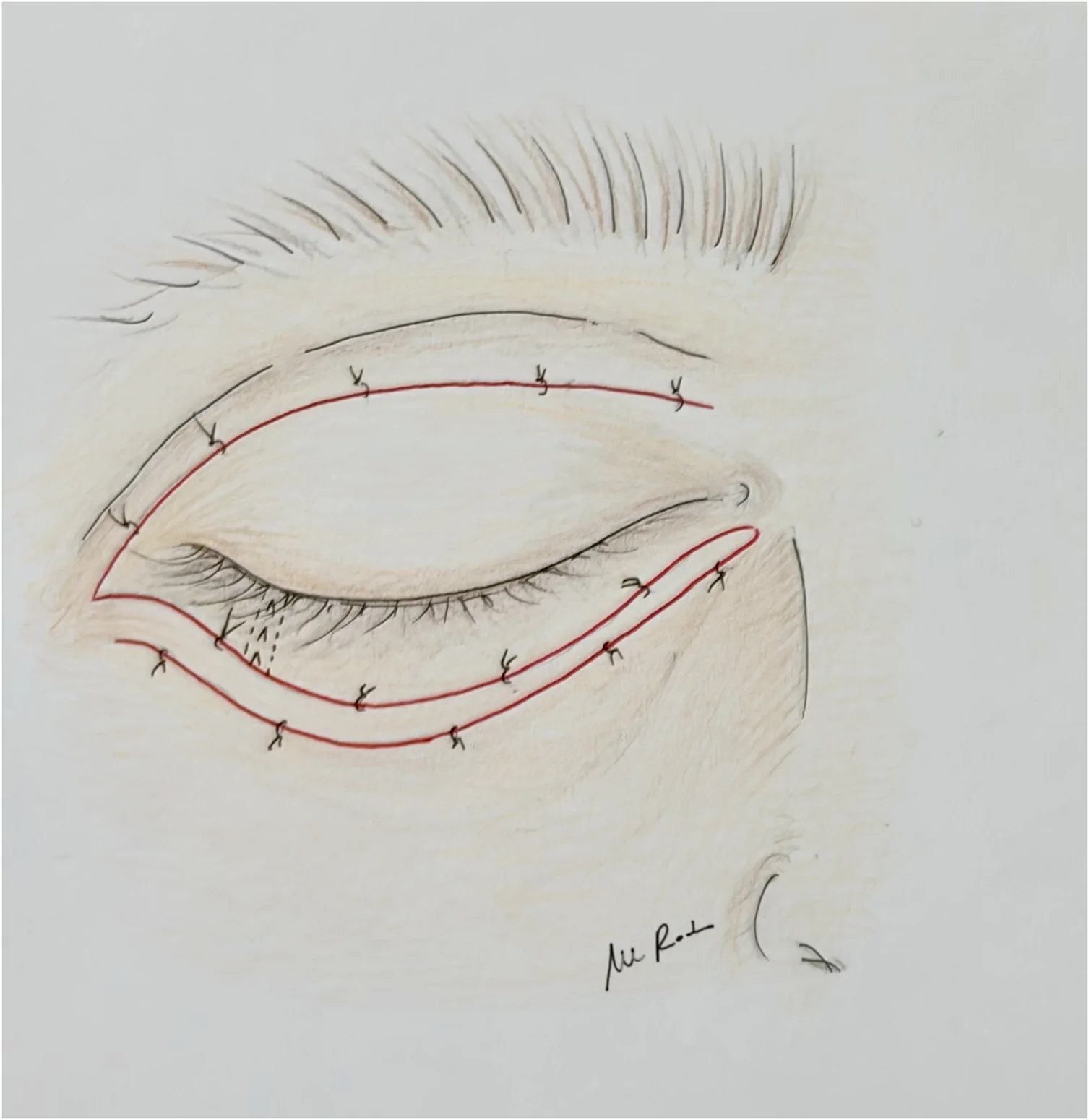
Transposition and securement of the upper eyelid myocutaneous flap. Reconstruction of the lower eyelid defect using the transposed upper eyelid flap. The flap, dissected with strict preservation of the lateral palpebral vessels and marginal arcade, is transposed inferiorly to the lower eyelid defect and anchored with interrupted 5-0 nylon sutures. Tarsal reapposition is achieved with a continuous 5-0 nylon suture, and the orbicularis layer is reapproximated using buried interrupted 5-0 Vicryl Rapid sutures.

**Figure 4:**
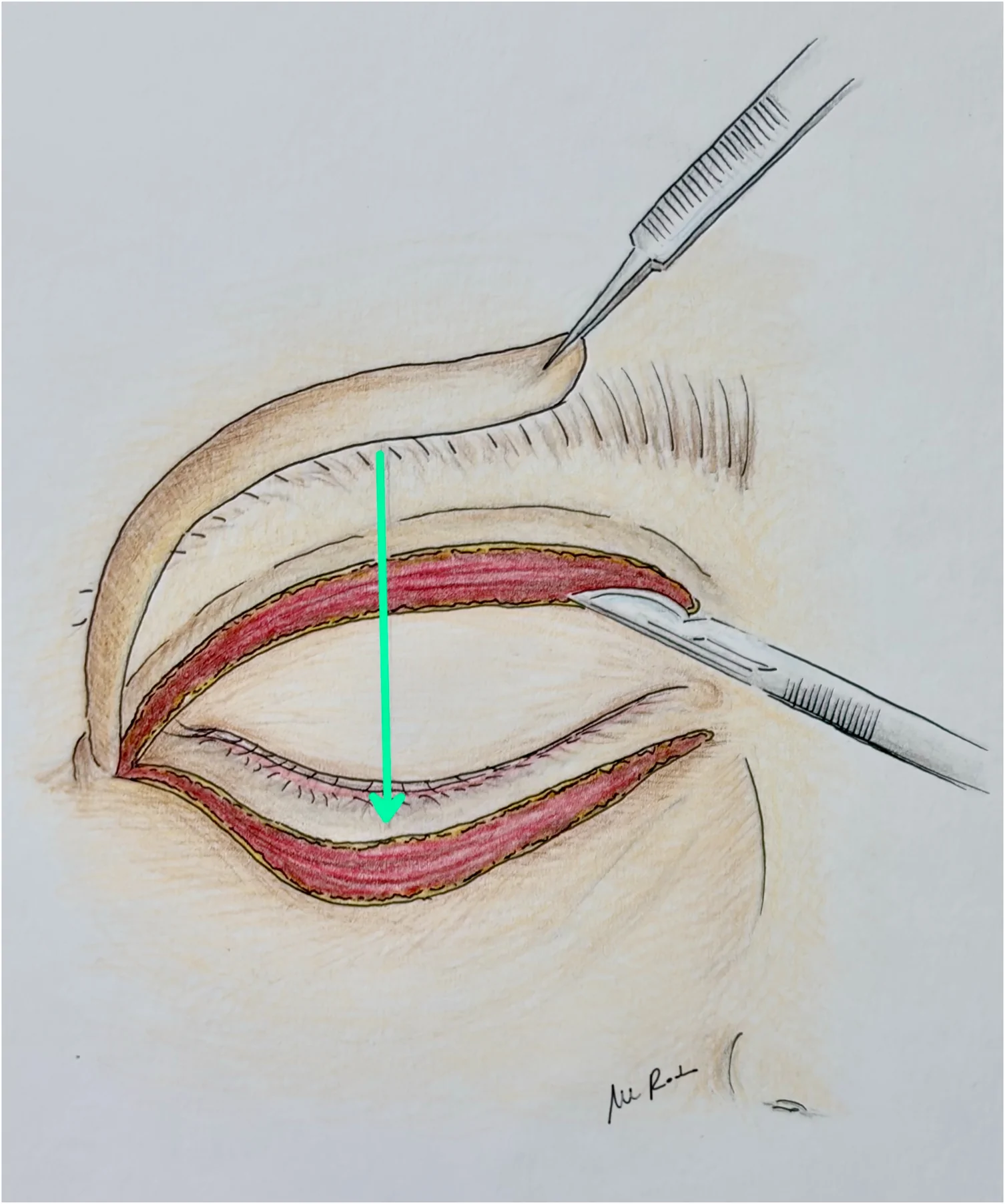
Primary closure of the upper eyelid donor site. Final operative result depicting primary closure of the upper eyelid donor defect within the supratarsal crease using interrupted 5-0 nylon sutures, restoring upper lid contour while providing tissue coverage for the lower eyelid reconstruction.

For entropion correction, a similar subciliary incision was performed 2 mm below the lower eyelid margin, followed by inferior and superior dissection of the skin from the underlying orbicularis oculi muscle for approximately 5–8 mm depending on individual anatomy (Figure 5). The prominent pretarsal orbicularis roll was identified and partially excised to expose the anterior tarsal surface, thereby reducing the inward rotational vector (Figure 6). Horizontal instability was corrected using a full-thickness base-down triangular resection at the lateral canthus; after an initial incision approximately 2 mm from the lateral canthal angle, the eyelid was laterally distracted to determine the appropriate width of resection (Figure 7) The tarsus was closed with a continuous 5-0 nylon suture placed through its deep portion, while the orbicularis layer was reapproximated using buried interrupted 5-0 Vicryl Rapid sutures. A strip of lower eyelid skin measuring approximately 1–3 mm was then excised from the inferior skin flap to provide an additional everting and stabilizing effect, and the skin was closed with interrupted 5-0 nylon sutures (Figure 8). At the end of surgery, antibiotic ointment (tobramycin 0.3%) was applied and an eye patch was placed.

- Step 1 – Orbicularis myectomy
- Step 2 – Base-down wedge resection
- Step 3 – Skin-strip excision

**Figure 5:**
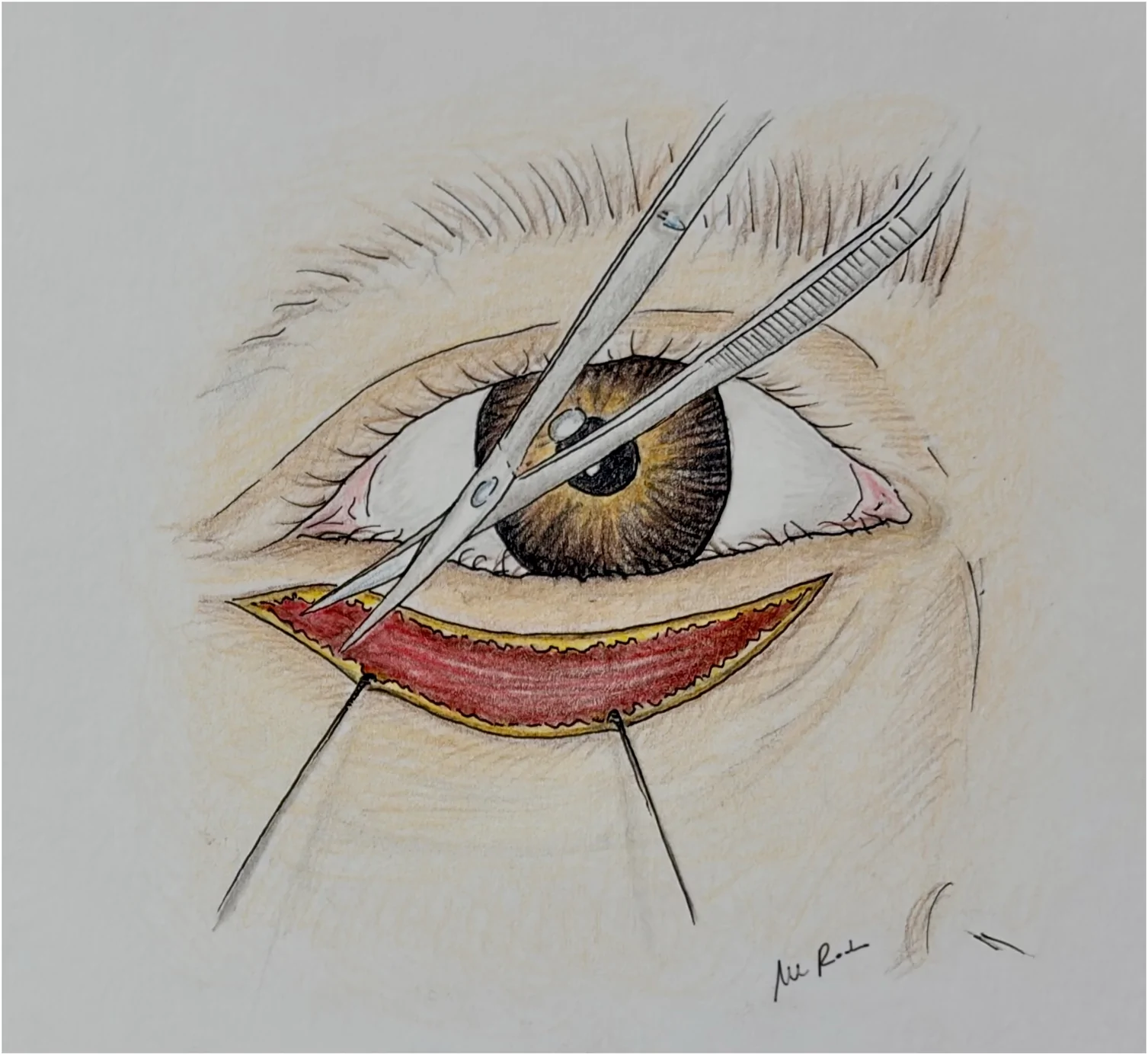
Subciliary incision and skin–orbicularis flap elevation for entropion correction. Intraoperative view showing the subciliary approach initiated 2 mm below the lower eyelid margin. Subcutaneous dissection is extended inferiorly and superiorly across a 5–8 mm zone to broadly liberate the skin from the underlying orbicularis oculi muscle, exposing the tarsal architecture.

**Figure 6:**
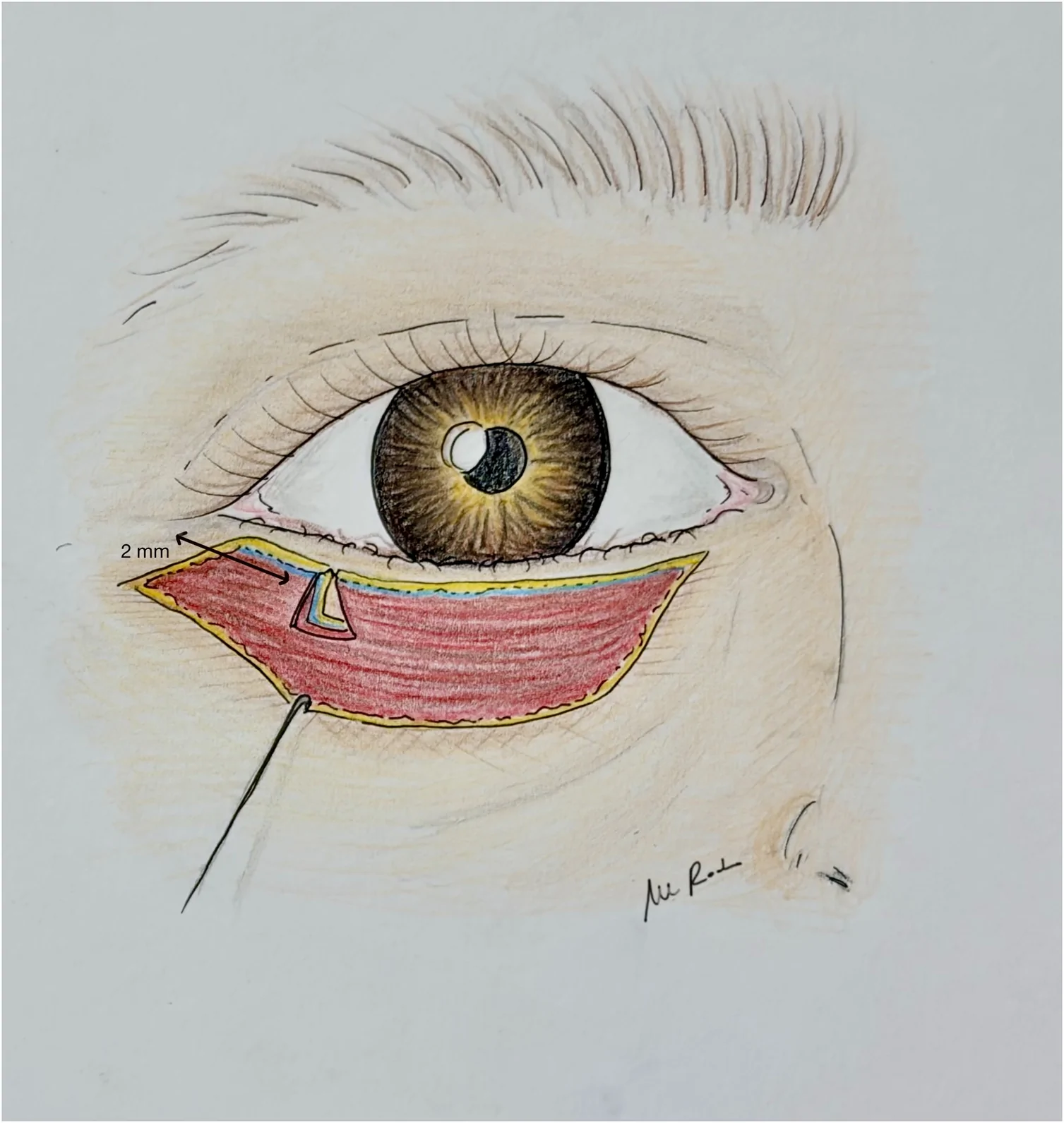
Partial excision of the pretarsal orbicularis roll. Targeted resection of the hypertrophic/prominent pretarsal orbicularis muscle fibers overlying the anterior tarsal surface. This maneuver neutralizes the pathological inward rotational vector driving eyelid inversion and restores normal margin position.

**Figure 7:**
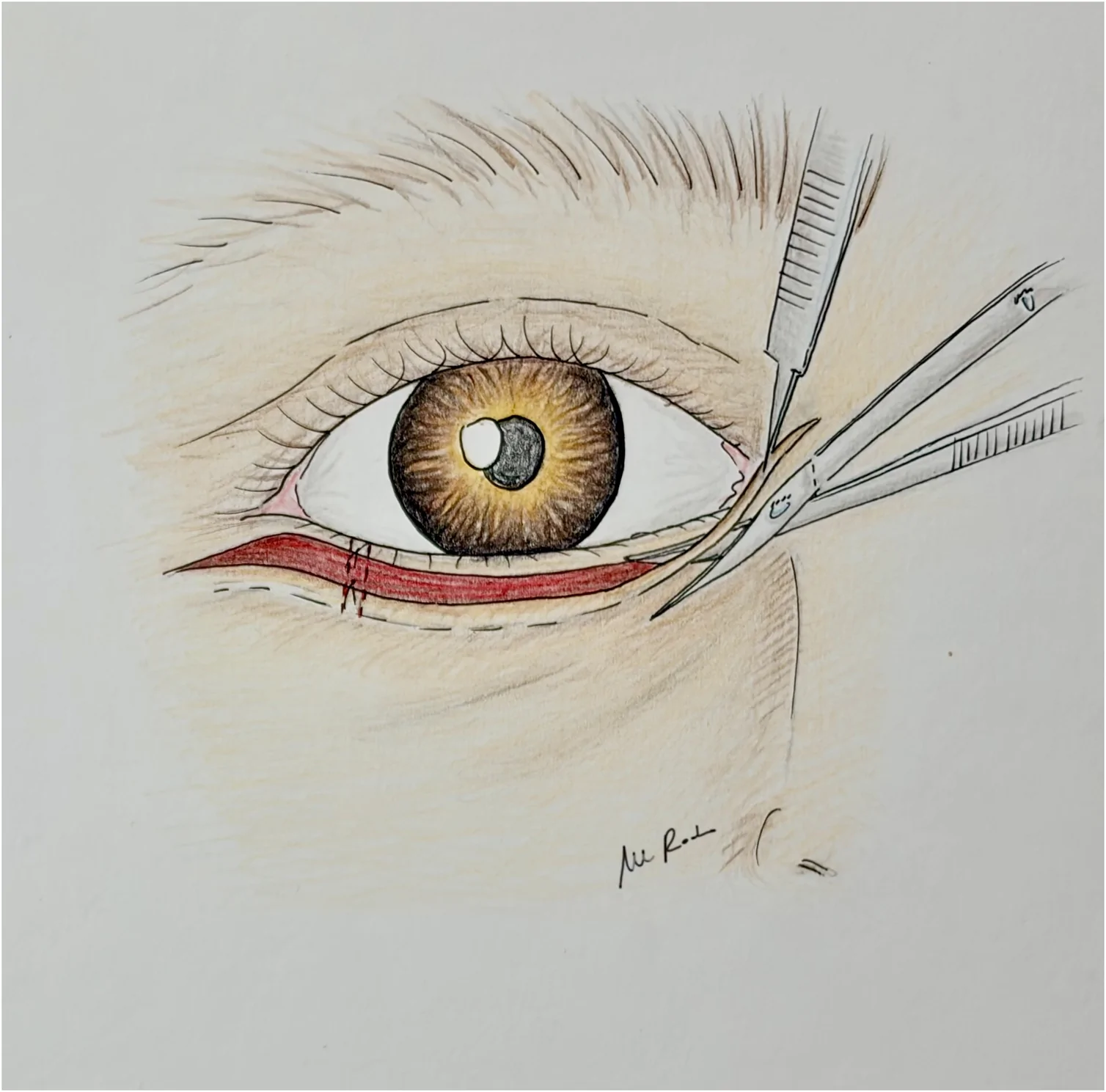
Correction of horizontal instability via full-thickness base-down triangular resection. Management of lower eyelid horizontal laxity at the lateral canthus. Following a primary incision placed 2 mm from the lateral canthal angle, the eyelid margin is distracted laterally to assess tissue redundancy. A full-thickness, base-down triangular resection is performed to stabilize the eyelid structure prior to closure.

**Figure 8:**
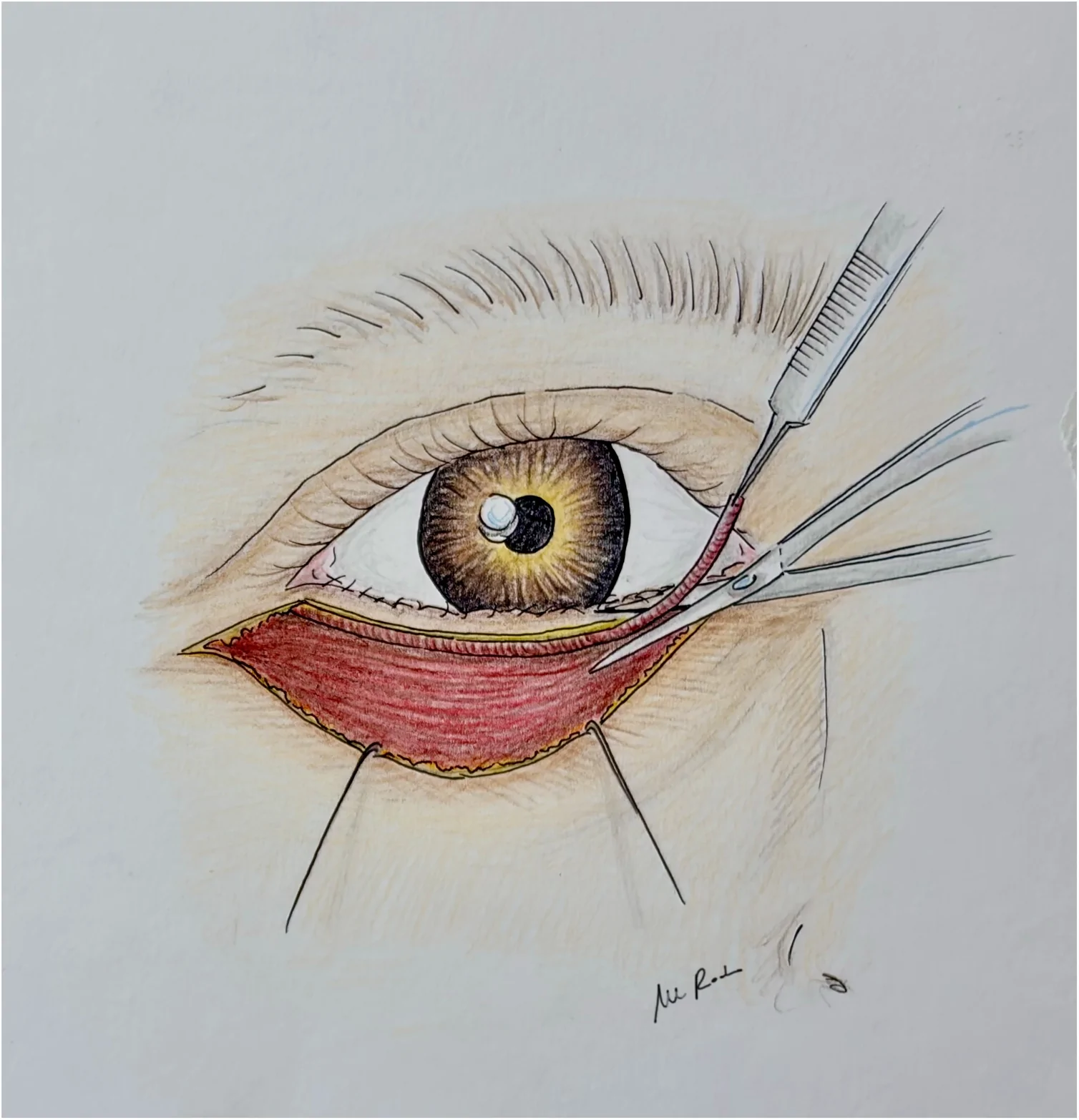
Tarsal closure, skin strip excision, and final everting skin reapposition. Final surgical steps and closure scheme. Deep tarsal reapproximation is executed with a continuous 5-0 nylon suture, complemented by buried interrupted 5-0 Vicryl Rapid sutures for the orbicularis layer. Excision of a 1–3 mm skin strip from the inferior flap margin delivers a secondary everting vector, with final skin closure completed using interrupted 5-0 nylon sutures.

Postoperative management in both techniques included topical tobramycin drops and ointment for two weeks, systemic analgesics, and lubricants, with skin sutures removed at 5–7 days and tarsal/marginal sutures removed at 7–10 days according to wound healing. Non-absorbable nylon was selected to provide stable tarsal approximation throughout the healing period and was routinely removed between postoperative days 7 and 10.

## Results

A total of 45 patients (57 eyelids) underwent surgical correction of involutional lower eyelid ectropion, while 35 patients (40 eyelids) underwent correction of involutional lower eyelid entropion using the standardized 3-step techniques.

Demographic and clinical characteristics are summarized in Table 2.

**Table 2:** Demographic characteristics, perioperative data, and outcomes of patients treated with 3-step procedures for involutional lower eyelid malpositions.

Demographic characteristics	Involutional ectropion	Involutional entropion
Patients, n	45	35
Eyelids, n	57	40
Male/Female	39/6	17/18
Age (years)	Median 77	Median 74
Laterality	Right 19 (42.2%) Left 14 (31.1%) Bilateral 12 (26.7%)	Unilateral 30 (85.7%) Bilateral 5 (14.3)%
Follow-up	Mean 22 months	Median 12 months
Surgical time	Mean 40 min (35–45)	Mean 37 min (30–42)
Early complications	1 wound dehiscence	1 wound dehiscence
Hematoma/infection	None	None
Undercorrection	1 case	1 case
Overcorrection	None	None
Late recurrence	2 cases	None
Overall success rate	94.7%	97.5%

The ectropion cohort included predominantly male patients, with a median age of 77 years (range 55–92). The entropion group had a median age of 74 years, with a slightly higher proportion of female patients. In the present ectropion cohort, 33 patients (73.3%) had unilateral disease, including 19 right-sided and 14 left-sided cases, whereas 12 patients (26.7%) had bilateral involvement, corresponding to a total of 57 treated eyelids.

The mean follow-up period was 22 months for the ectropion cohort, whereas the median follow-up period was 12 months for the entropion cohort. In both cohorts, no cases of postoperative hematoma or infection were observed. One case of early wound dehiscence occurred in each group, both involving the tarsal incision and successfully managed with resuturing.

Forty-five patients (57 eyelids) underwent ectropion correction, with a mean follow-up of 22 months. The overall success rate was 94.7% (54/57 eyelids). One case of undercorrection and two late recurrences were observed, all successfully managed with secondary procedures. Complications were minimal and limited to one early wound dehiscence. (Figure 9).

**Figure 9:**
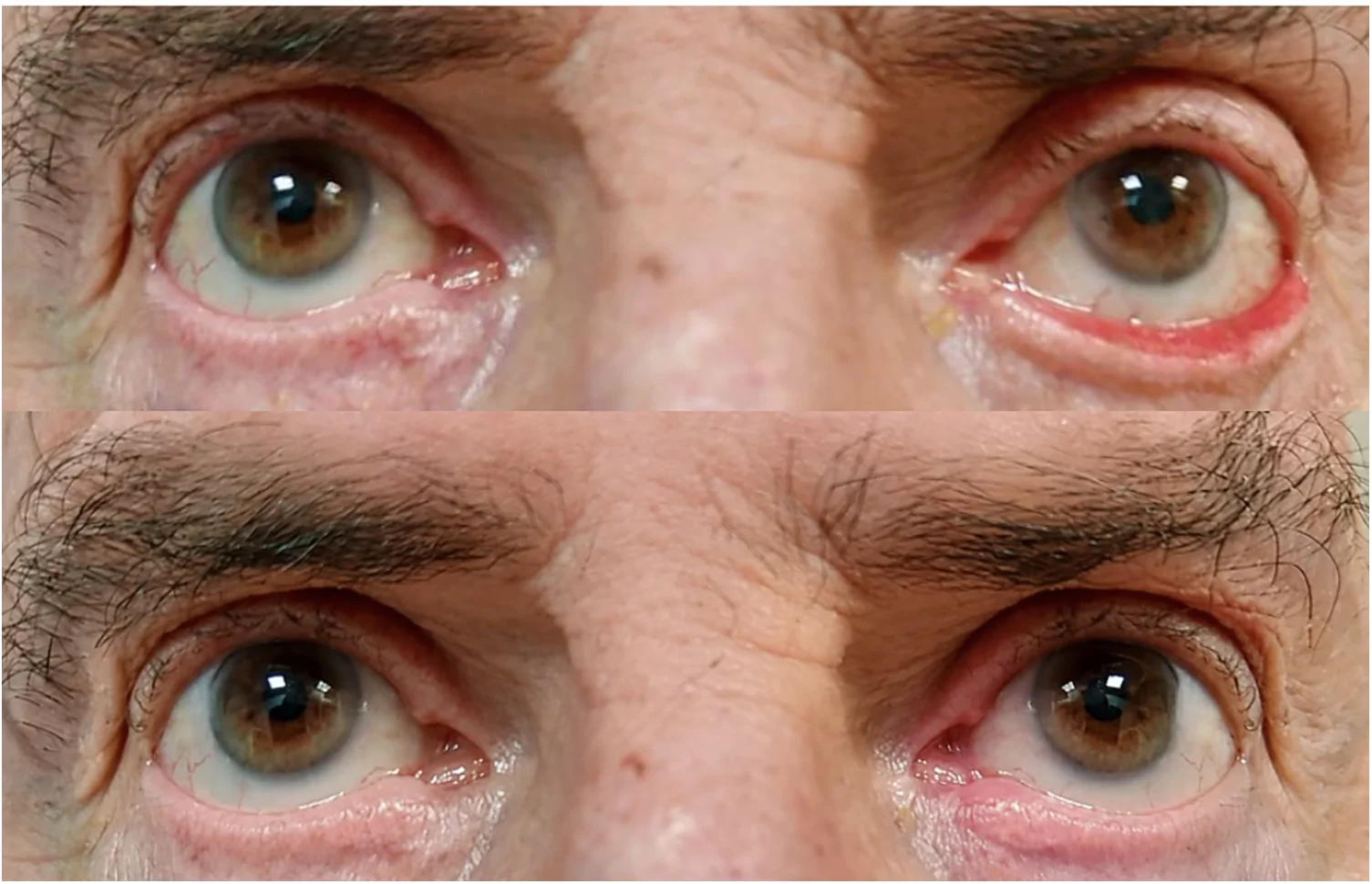
Eighty-five-year-old male patient with involutional ectropion of the left lower eyelid. Pre-operative picture showing a grade III ectropion with the exposure of the lower eyelid fornix. The post-operative picture at 12 months of follow-up showing a normal apposition of the lower eyelid to the ocular surface. The patient underwent the three-step ectropion procedure described in the present study. The patient belongs to the study cohort. The photographs have not been previously published, were obtained with appropriate written consent for publication, and have been adequately anonymized.

In the entropion group, one case of undercorrection was observed without lash–cornea contact and required no further intervention. No cases of overcorrection, recurrence, or late complications were reported. The overall success rate was 97.5% (39/40 eyelids). (Figure 10).

**Figure 10:**
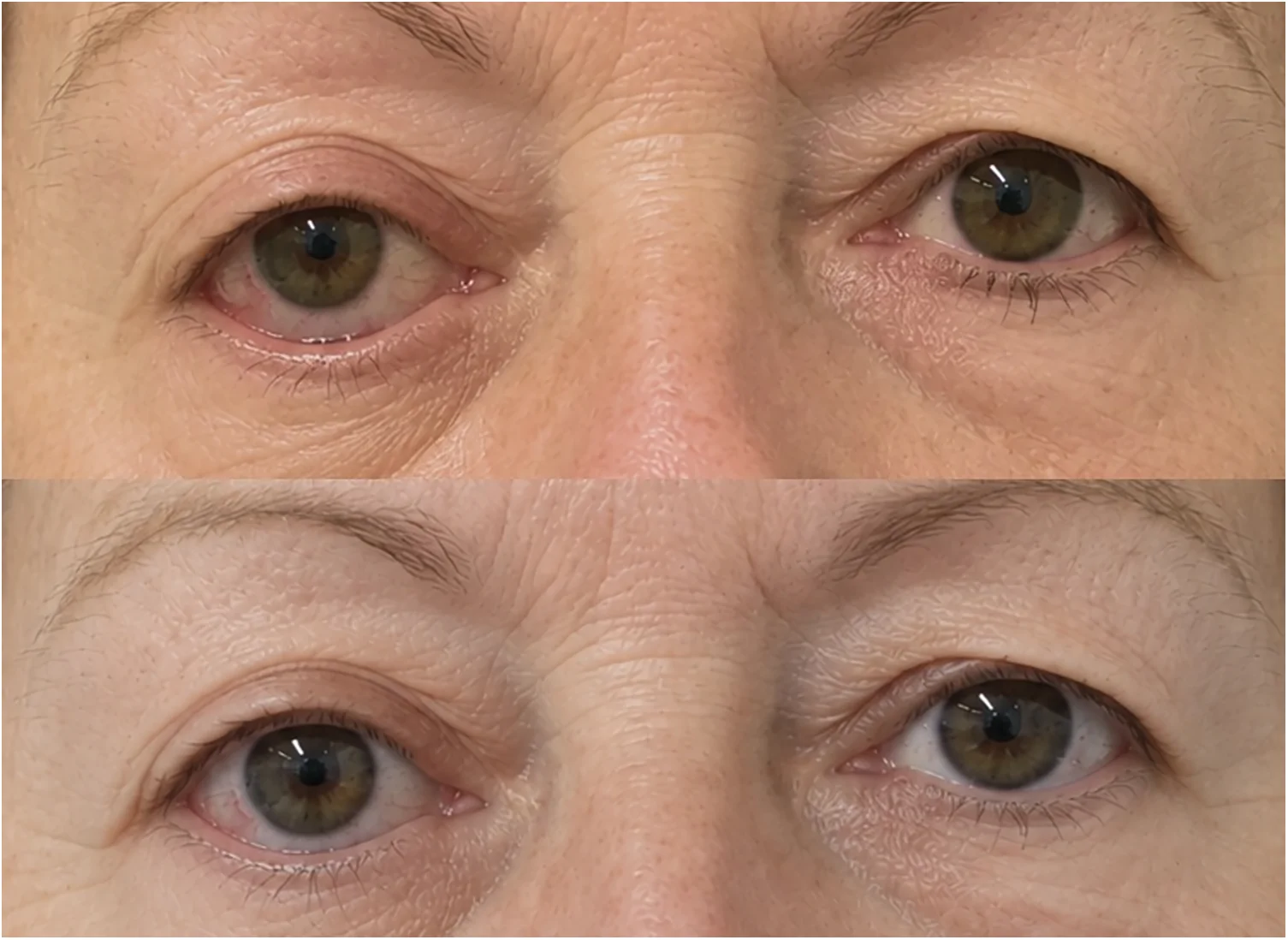
Seventy-year-old female patient with involutional entropion of the right lower eyelid. Pre-operative picture showing entropion of the lower eyelid, characterized by inward rotation of the lid margin. Post-operative picture showing the eyelashes directed outward and away from the ocular surface at rest at 12 months of follow-up. The patient underwent the three-step entropion procedure described in the present study. The patient belongs to the study cohort. The photographs have not been previously published, were obtained with appropriate written consent for publication, and have been adequately anonymized.

No cases of suture exposure, corneal irritation, or ocular surface complications related to the tarsal nylon sutures were observed during follow-up.

### Clinical contribution

- Previous: Initial evaluation of individual technique
- Present: Expanded cohort + updated follow-up + unified pathophysiological/vector-based interpretation

### Statistical analysis

- Previous: Descriptive analysis of individual cohort
- Present: Updated descriptive analysis of expanded cohorts; no comparative statistical testing between ectropion and entropion

## Discussion

Involutional lower eyelid malpositions represent a spectrum of age-related disorders resulting from progressive degeneration of the anatomical structures responsible for eyelid stability (18). Although ectropion and entropion manifest with opposite clinical presentations, both conditions share several age-related anatomical and biomechanical abnormalities, particularly horizontal laxity and altered lamellar and orbicularis forces (19)

This shared pathophysiological background provided the rationale for evaluating the two conditions within the same study. From a methodological perspective, the present investigation should not be interpreted as the simultaneous analysis of two unrelated disease entities. Rather, it was designed to evaluate a unified reconstructive concept applied to two opposite clinical manifestations of lower eyelid involution. The principal added value of the present study lies in the integration of the two previously described procedures within a common biomechanical and pathophysiological framework in which similar age-related structural abnormalities are corrected through condition-specific procedures that generate opposite restorative vectors. In this regard, ectropion and entropion represent complementary models for evaluating the effectiveness of the same pathophysiological and reconstructive principles.

These structural and functional alterations disrupt the delicate equilibrium of forces maintaining the lower eyelid in its physiological position, ultimately leading to either inward or outward rotation of the lid margin. Successful surgical correction therefore requires not only restoration of eyelid tension, but also rebalancing of lamellar forces and normalization of orbicularis muscle dynamics (11–20).

Numerous surgical procedures have been proposed for the treatment of involutional ectropion and entropion, including lateral tarsal strip (LTS), wedge resections, everting sutures, retractor reinsertion, skin-muscle excision, and orbicularis suspension techniques (21) (22) (23).

However, no single procedure has demonstrated clear superiority, and techniques addressing only one etiologic factor have generally shown higher recurrence rates.

For this reason, combined approaches targeting multiple pathogenic mechanisms have progressively gained popularity.

The individual three-step procedures evaluated in this study were previously described by our group in separate reports addressing involutional ectropion and entropion (15, 16). We acknowledge that the present study therefore does not introduce either surgical technique as a novel operative procedure. The relationship between the previously reported cohorts and the present study is instead characterized by cohort expansion and updated clinical assessment. The current ectropion cohort comprises 45 patients involving 57 eyelids, including the 36 patients and 45 eyelids reported previously, together with 9 additional patients involving 12 additional eyelids. Similarly, the current entropion cohort comprises 35 patients involving 40 eyelids, including the 27 patients and 30 eyelids previously reported, together with 8 additional patients involving 10 additional eyelids. Follow-up of the previously reported patients was updated in the present analysis.

The principal contribution of the present study is therefore not the introduction of a new surgical technique, but the integration of the two previously described procedures within a common pathophysiological and biomechanical framework. The previous publications primarily focused on the clinical application and outcomes of each individual procedure. In the present study, the two procedures are considered complementary, disease-specific strategies addressing opposite rotational manifestations of age-related lower eyelid instability. This framework emphasizes three shared reconstructive objectives: restoration of horizontal eyelid tension, re-establishment of anterior–posterior lamellar balance, and modulation of abnormal orbicularis-related forces.

This conceptual integration does not imply that ectropion and entropion are anatomically identical or that their surgical management is interchangeable. Rather, the procedures retain their condition-specific technical components and apply corrective vectors in opposite directions according to the direction of the pathological eyelid rotation. The ectropion procedure combines horizontal shortening with anterior lamellar release and a laterally based myocutaneous flap, whereas the entropion procedure combines horizontal shortening with orbicularis modification and limited anterior lamellar shortening. The common framework therefore lies in the reconstructive principles rather than in the operative steps themselves.

From a clinical perspective, the present study extends the available experience with both procedures by incorporating additional patients and updated follow-up of the previously reported cohorts. From a methodological perspective, the study applies the same predefined clinical eligibility criteria, outcome definitions, and complication assessment to the two current cohorts, allowing their results to be interpreted within a consistent observational framework. No formal comparative statistical analysis between ectropion and entropion was performed because the two conditions have different clinical manifestations, outcome definitions, and disease-specific surgical objectives.

Despite their technical differences, both procedures are founded on the same reconstructive principles. The fundamental step in both techniques is horizontal eyelid shortening through wedge resection. Horizontal laxity is widely recognized as the primary pathogenic factor in involutional lower eyelid malpositions (5–24), as elongation of the eyelid disrupts the apposition between the lid and the globe, predisposing the eyelid to rotate either inward or outward (25, 26).

In ectropion, a base-up triangular resection was performed to generate an upward and inward vector capable of repositioning the everted eyelid against the globe. Conversely, in entropion, a base-down triangular resection was used to rotate the inferior portion of the tarsus outward and restore the physiological orientation of the lid margin and eyelashes.

Several authors have advocated the use of the LTS procedure for correcting horizontal laxity (8) (8); however, we prefer wedge resection techniques because they preserve the integrity of the lateral canthal tendon and reduce the risk of lateral canthal angle distortion, wound dehiscence, and granuloma formation (27). Nevertheless, wedge resection is not without limitations. Because the procedure requires full-thickness interruption of the tarsus and posterior lamella, a theoretical risk of eyelid margin weakening exists, particularly in cases of wound dehiscence. Therefore, meticulous tarsal closure is essential to restore structural continuity. In our series, although one wound dehiscence occurred in each group, both cases were successfully managed with resuturing and no long-term eyelid instability or margin deformity was observed.

The two procedures diverge according to the specific direction of eyelid imbalance. In involutional ectropion, horizontal eyelid laxity remains the principal pathogenic mechanism. However, in longstanding cases, chronic exposure, recurrent inflammation, loss of orbicularis support, age-related midface descent, and secondary skin–muscle adhesions may further contribute to the outward rotational vector and impair lower eyelid apposition. For this reason, our technique combines horizontal shortening with release of the anterior lamella and reconstruction using a laterally based upper eyelid myocutaneous flap. Beyond replacing tissue after release, the flap provides additional vertical support and an upward suspensive effect that contributes to stabilization of the corrected eyelid position (28).

In contrast, involutional entropion is characterized by overriding of the preseptal OOM over the pretarsal portion, generating an inward rotational force that turns the eyelashes toward the globe. For this reason, the entropion procedure included partial myectomy of the preseptal OOM to eliminate the inward vector responsible for eyelid inversion. The final excision of a small strip of lower eyelid skin acted as an everting maneuver by shortening the anterior lamella and stabilizing the corrected lid position. Careful calibration of skin excision proved essential to avoid overcorrection and postoperative ectropion (29, 30).

The results of the present study support the effectiveness and reliability of a pathophysiology-driven combined approach. In the ectropion group, the overall success rate was 94.7% (54/57 eyelids), with one case of undercorrection and two late recurrences. All three cases were successfully managed with secondary procedures. The occurrence of two late recurrences despite an overall favorable outcome also supports the importance of prolonged postoperative surveillance in patients undergoing correction of involutional ectropion. In the entropion group, no recurrences were observed during follow-up, and only one mild undercorrection occurred without symptomatic trichiasis or need for revision surgery. In both groups, postoperative complications were minimal and limited to isolated cases of early wound dehiscence. No cases of infection, hematoma, scar contracture, eyelid notching, chronic ocular irritation, or overcorrection were reported. No validated patient-reported outcome measures (PROMs) were collected in this retrospective cohort. Although subjective symptom improvement and patient satisfaction were occasionally documented during routine postoperative visits, these assessments were not collected using a standardized questionnaire or formal rating scale and therefore cannot be considered validated measures of patient-reported benefit.

Reported success rates for lateral tarsal strip procedures generally range from approximately 80% to 95% (8), with recurrence rates varying according to follow-up duration and patient selection. Wedge resection techniques have demonstrated comparable outcomes, while isolated procedures such as everting sutures or retractor reinsertion may be associated with higher recurrence rates when performed alone. More recently, combined procedures addressing multiple pathogenic mechanisms have reported success rates exceeding 90% (25). The outcomes observed in our series (94.7% for ectropion and 97.5% for entropion) therefore appear consistent with those reported for contemporary multi-component approaches.

Our findings are consistent with previous studies demonstrating improved long-term outcomes when multiple anatomical factors are addressed simultaneously. Combined techniques described by Ranno et al. (10), Abdullah et al. (31), and other authors similarly reported reduced recurrence rates compared with isolated procedures targeting single etiologic components. However, unlike more invasive combined approaches requiring canthotomy, cantholysis, or extensive lower eyelid retractor dissection, the procedures described in this study remain relatively conservative while still comprehensively addressing the underlying biomechanical abnormalities.

An additional strength of these techniques lies in their conceptual symmetry. Although involutional ectropion and entropion are not exact anatomical mirror images, both arise from age-related degeneration of the lower eyelid support structures and share several pathogenic factors, including horizontal laxity, orbicularis dysfunction, and alterations in lamellar balance. The complementary vector-based concept proposed in this study refers primarily to eyelid biomechanics and vector correction. While entropion results from an inward rotational vector and ectropion from an outward rotational vector, both procedures are designed to neutralize these opposing forces through analogous reconstructive principles. This concept is particularly reflected in the opposite orientation of the triangular wedge resection and in the direction of the corrective vectors applied to restore physiological eyelid position (15) Accordingly, the two procedures share the same reconstructive philosophy while applying opposite corrective forces: inward and upward stabilization in ectropion, outward rotation and eversion in entropion. This parallel approach highlights the importance of understanding lower eyelid biomechanics when planning surgical correction (32).

The present study has several limitations. First, its retrospective nature and the absence of a control group preclude direct comparison with alternative surgical techniques. Second, the sample size remains relatively limited despite representing one of the larger single-center experiences with these combined procedures.

An additional important limitation is the absence of standardized patient-reported outcome measures (PROMs). No validated questionnaires or formal patient-reported rating scales were administered in the present retrospective cohort. Although subjective symptom improvement and patient satisfaction were occasionally documented during routine postoperative visits, these assessments were not standardized and therefore cannot be considered validated measures of patient-reported benefit. This limitation is particularly relevant in the evaluation of functional and aesthetic eyelid surgery. Future prospective studies should incorporate validated PROMs, such as FACE-Q or disease-specific questionnaires, to provide standardized and quantitative assessment of functional symptoms, aesthetic satisfaction, and quality of life.

Furthermore, our cohort consisted predominantly of Caucasian patients. Anatomical differences among ethnic groups, particularly regarding upper eyelid crease morphology and scar characteristics, may influence the applicability and aesthetic outcomes of the upper eyelid flap technique. Further studies in more diverse populations are warranted. Finally, follow-up duration was heterogeneous, particularly in the entropion cohort, reflecting the difficulty of maintaining long-term surveillance in patients affected by benign eyelid conditions who often become asymptomatic after surgery.

Overall, our results suggest that a tailored, pathophysiology-based reconstructive strategy provides safe, effective, and durable correction of involutional lower eyelid malpositions. By simultaneously correcting lamellar imbalance, restoring horizontal tension and normalizing orbicularis muscle forces, these 3-step procedures achieve stable anatomical and functional outcomes with low complication and recurrence rates. Future prospective comparative studies with larger cohorts and standardized long-term follow-up are warranted to further validate these findings and better define the role of these techniques within the surgical management of involutional lower eyelid malpositions.

## Conclusion

Involutional ectropion and entropion are clinically distinct lower eyelid malpositions that share several age-related biomechanical abnormalities, including horizontal eyelid laxity, lamellar imbalance, and altered orbicularis oculi dynamics. The present study does not introduce new surgical procedures, but provides an expanded and updated clinical evaluation of two previously described, condition-specific three-step techniques within a unified reconstructive framework.

Although the operative steps differ according to the direction of eyelid malposition, both procedures pursue the same fundamental objectives: restoration of horizontal eyelid support, rebalancing of the eyelid lamellae, and correction of abnormal rotational forces. The favorable outcomes observed in the present expanded cohorts support this pathophysiology-driven and vector-based approach. Prospective studies with standardized outcome measures and longer follow-up are warranted to further validate these findings.

## Data Availability

The original contributions presented in the study are included in the article/supplementary material, further inquiries can be directed to the corresponding author.
